# Ribosome profiling uncovers selective mRNA translation associated with eIF2 phosphorylation in erythroid progenitors

**DOI:** 10.1371/journal.pone.0193790

**Published:** 2018-04-10

**Authors:** Nahuel A. Paolini, Kat S. Moore, Franca M. di Summa, Ivo F. A. C. Fokkema, Peter A. C. ‘t Hoen, Marieke von Lindern

**Affiliations:** 1 Department of Hematopoiesis, Sanquin Research, and Landsteiner Laboratory AMC/UvA, Amsterdam, The Netherlands; 2 Department of Human Genetics, Leiden University Medical Center, Leiden, The Netherlands; 3 Centre for Molecular and Biomolecular Informatics, Radboud Institute for Molecular Life Sciences, Radboud University Medical Center, Nijmegen, The Netherlands; University of British Columbia, CANADA

## Abstract

The regulation of translation initiation factor 2 (eIF2) is important for erythroid survival and differentiation. Lack of iron, a critical component of heme and hemoglobin, activates Heme Regulated Inhibitor (HRI). This results in phosphorylation of eIF2 and reduced eIF2 availability, which inhibits protein synthesis. Translation of specific transcripts such as *Atf4*, however, is enhanced. Upstream open reading frames (uORFs) are key to this regulation. The aim of this study is to investigate how tunicamycin treatment, that induces eIF2 phosphorylation, affects mRNA translation in erythroblasts. Ribosome profiling combined with RNA sequencing was used to determine translation initiation sites and ribosome density on individual transcripts. Treatment of erythroblasts with Tunicamycin (Tm) increased phosphorylation of eIF2 2-fold. At a false discovery rate of 1%, ribosome density was increased for 147 transcripts, among which transcriptional regulators such as *Atf4*, *Tis7/Ifrd1*, *Pnrc2*, *Gtf2h*, *Mbd3*, *JunB* and *Kmt2e*. Translation of 337 transcripts decreased more than average, among which *Dym* and *Csde1*. Ribosome profiling following Harringtonine treatment uncovered novel translation initiation sites and uORFs. Surprisingly, translated uORFs did not predict the sensitivity of transcripts to altered ribosome recruitment in presence or absence of Tm. The regulation of transcription and translation factors in reponse to eIF2 phosphorylation may explain the large overall response to iron deficiency in erythroblasts.

## Introduction

Mature erythrocytes contain approximately 2.5x10^8^ hemoglobin molecules per cell, each existing of 4 globin polypeptides associated with an iron loaded heme molecule. The synthesis of heme and globin must be tightly balanced to prevent proteotoxic stress caused by an excess of iron or free globins [1]. The Iron response element binding proteins Irp1 (Aco1) and Irp2 (Ireb2) control mRNA stability and translation of transcripts encoding proteins involved in iron homeostasis such as the Transferrin receptor, Ferroportin, and Ferritin [2]. In addition, mechanisms to prevent proteotoxicity in general are crucial in erythropoiesis. Proteotoxic stress leads to activation of kinases that phosphorylate the alpha subunit of translation initiation factor 2 (eIF2α) to inhibit translation. The four eIF2α kinases are HRI (heme regulated inhibitor, or Eif2ak1) that is activated by oxidative stress or lack of heme, the double-stranded RNA-dependent kinase (PKR, or Eif2ak2), the endoplasmic reticulum (ER) stress activated kinase PERK (Eif2ak3) and GCN2 (general control nonderepressible 2 or Eif2ak4) that is activated by uncharged tRNA upon lack of amino acids [3].

GTP-bound eIF2 and methionine-loaded initiatior tRNA (tRNA_i_^met^) form the ternary complex (TC). The TC binds to the 40S small ribosomal subunit in the preinitiation scanning complex. The GTPase activity of eIF2 is activated when the scanning complex pauses at a translation start site, which results in release of methionine to the P-site of the ribosome, and dissociation of both tRNA_i_ and GDP-bound eIF2 from the scanning complex [4]. The GDP-GTP exchange factor eIF2B reloads eIF2 with GTP, which enables eIF2 to bind tRNA_i_^met^ and to re-associate with a preinitiation scanning complex. Phosphorylation of the α-chain of eIF2 (eIF2α) on Ser51 by HRI prevents exchange of GDP for GTP and thereby recovery of the TC. As a result protein synthesis is inhibited to decrease for instance globin production, which prevents damage from globin protein aggregates [5].

Translational control by eIF2 is, at least in part, mediated through translation of upstream open reading frames (uORFs). Whereas general translation is repressed, translation of specific transcripts is increased upon eIF2 phosphorylation, as described for *Atf4*. A distance of ~90 nt between the first and second uORF allows for re-association in absence of eIF2 phosphorylation [6]. Translation of the second uORF overlapping the start codon of the protein coding ORF inhibits Atf4 protein expression. Reduced availability of eIF2 decreases translation initiation at the second uORF (also referred to as leaky scanning), and increases translation of the *Atf4* protein coding ORF. The short distance between uORFs is crucial for eIF2-mediated control of translation [6,7]. Phosphorylation of eIF2 also reduces the recognition of start codons in a suboptimal Kozak consensus context as is exemplified by the regulation of *Ddit3* (Death and differentiation induced transcript 3, also known as Chop). The inhibitory uORF of *Ddit3* is poorly translated upon eIF2 phosphorylation, which increases Ddit3 protein expression [8]. Depending on the configuration of the 5´UTR, translation of specific transcripts can also be hypersensitive for eIF2 and cause a more than average repression of translation, as has been described for *Csde1* [9].

Whereas these examples demonstrate quantitative effects on protein synthesis, uORFs are also involved in qualitative changes in protein expression. A short distance between an uORF and the start codon of the protein coding ORF may result in partial availability of the protein initiating start codon. The presence of a downstream, in frame, start codon can subsequently result in expression of an N-terminally truncated short isoform. This leaky scanning controls for instance the balance between the long and short isoform of Tal1/Scl, an important transcription factor in erythropoiesis [10].

Heme-regulated phosphorylation of eIF2 and the subsequent regulation of mRNA translation, is important in the control of erythropoiesis. HRI-induced expression of Atf4 and its downstream target Ppp1r15a/Gadd34 constitutes an integrated stress response (ISR) that increases survival of erythroid cells when mice are fed a low iron diet [11]. Atf4 null mice displayed severe fetal anemia [12]. Modulation of the ISR response is regulated by the dephosphorylation of eIF2 by Ppp1r15a and Ppp1r15b [13,14]. Loss of Ppp1r15a results in enlarged spleens with increased numbers of immature erythroid cells and low hemoglobin content [15]. Loss of Ppp1r15b increases the number of deformed erythroblasts and reduces the number of mature erythrocytes. The erythrocyte numbers were rescued when loss of Ppp1r15b was combined with the S51A knock-in mutation of eIF2, that abrogates eIF2 phosphorylation [16]. These phenotypes indicate that eIF2 phosphorylation is important for control of both expansion and differentiation of erythroblasts. Animal models for iron deficiency anemia indicate that not only differentiation, but also expansion of immature erythroblasts is impaired [17]. The cellular mechanism responsible for impaired erythropoiesis upon iron deficiency, however, is poorly understood.

Polyribosome profiling has established selective mRNA translation in erythropoiesis [18,19]. Ribosome footprinting or ribo-seq allows for deep sequencing of mRNA fragments protected by the ribosome (ribosome footprints, RFPs) [20,21]. The RFPs are aligned to the genome, which maps the position of ribosomes at the nucleotide level and adds considerable detail to the analysis of mRNA translation. The aim of this study is to identify transcripts that are hypersensitive to eIF2 phosphorylation in erythroblasts. We hypothesize that translation of uORFs renders transcripts sensitive to eIF2 phosphorylation because it controls re-association of the TC with the preinitiation scanning complex, which is required for translation of a subsequent ORF. We aim to identify cellular mechanisms regulated by eIF2 phosphorylation that are involved in erythroid homeostasis. We employed ribosome footprint analysis in combination with mRNA sequencing to identify both translation initiation sites (TIS) and the relative translation efficiency of transcripts. At a false discovery rate (FDR) of 1% we identified 147 transcripts subject to increased ribosome density, and 337 transcripts subject to reduced ribosome density upon treatment of erythroblasts with Tunicamycin, a drug that efficiently induces eIF2 phosphorylation. Interestingly, translation of uORFs was widespread, but did not predict sensitivity of the mRNA translation to eIF2 phosphorylation. Among the transcripts subject to eIF2-dependent translation were several transcription factors that may alter programming of erythropoiesis upon eIF2 phosphorylation.

## Materials and methods

### Cell culture

The erythroblast cell line 15.4 was derived from p53-deficient mouse fetal livers as previously described [22], and cultured in Stempro-34 SFM (Thermo Fisher), containing penicilin-streptavidin, L-glutamin, Erythropoietin (1U/ml), Stem Cell Factor (supernatant CHO cells) and 1μM Dexamethasone (Sigma) [23]. For ER stress induction, cells were treated with 2.5μg/ml Tunicamycin (Tm) (Sigma) for 1.5h or left untreated.

### SDS-PAGE

Whole cell lysates were loaded on 10% polyacrylamide gels (Biorad). Western blots were performed as previously described [18]. Antibodies used were eIF2 (Cell Signaling) and pSer51-eIF2 (Cell Signaling).

### Polysome profiling

10^7^ cells were lysed in polysome lysis buffer (110 mM potassium acetate, 20 mM magnesiumacetate, 10 mM HEPES, 100 mM potassium chloride, 10 mM magnesium chloride, 0.1% NP-40, 2 mM DTT, 40 U/mL RNase inhibitor [Thermo Fisher], 100 μg/ml cycloheximide [CHX] [Sigma] and 1X mini Protease Inhibitor Cocktail [Roche]) and loaded onto 17–50% sucrose gradients [24]. The tubes were centrifuged at 40,000 rpm for 2 hours at 4°C in a SW41 rotor (Optima L100XP ultracentrifuge; Beckman Coulter). RNA was measured throughout the gradient with a BR-188 Density Gradient Fractionation System at OD_254_ (Brandel). Area under the curve was calculated with Fiji, statistical significance was calculated with a t-test. P-values < 0.01 were considered significant.

### Measurement of de novo protein synthesis

100,000 erythroblasts were seeded in methionine-free DMEM (Invitrogen) for 60 minutes to deplete intracellular methionine, followed by a 90 minutes exposure to Click-iT^®^ AHA (a methionine analogue) in absence or presence of 2.5μg/ml Tm treatment. Newly synthesised protein was measured using the Click-iT^®^ AHA Alexa Fluor^®^ 488 Protein Synthesis HCS Assay (Thermo Scientific) according to manufacturer’s instructions with some modifications (2% paraformaldehyde for fixation and 1:1000 dilution of AHA). Fluorescence was measured by using an LSR-II flow cytometer and analyzed with FACSDiva software (BD Biosciences).

### Ribosome profiling and RNAseq

The ribosome profiling strategy was adapted from Ingolia et al. [25] and based on De Klerk et al. [26], with some modifications. After Tm treatment, 40*10^6^ cells were collected in 1 ml medium, treated with 100 μg/ml cycloheximide (CHX) for 5 min at 37 °C or 2 μg/ml Harringtonine for 7 min followed by 2 min 100 μg/ml CHX at 37 °C. Cells were washed wit ice-cold PBS, and lysed in 1 ml polysome lysis buffer. Lysates were treated with 1500 units of RNAse-I (Ambion) to digest the polysomes into monosomes. The 80S monosome fraction was isolated by ultracentrifugation (Beckman) on sucrose gradients and RNA was isolated as described [26]. Ribosomal RNA (rRNA) was removed with Ribozero Gold rRNA Removal Kit (Illumina). In this study, the sequencing library was made with Nebnext small RNA Library Prep Set for Illumina (NEB), according to manufacturer’s instructions, and the library was sequenced on a HiSeq Illumina (Leiden Genome Technology Center (LGTC), LUMC, Leiden, The Netherlands). For RNAseq, mRNA was isolated, reverse transcribed using oligodT primers, cDNA was fragmented transferred into a library and sequenced on a Hiseq Illumina using the Truseq protocol Illumina (Leiden Genome Technology Center (LGTC), LUMC, Leiden, The Netherlands).

### Data analysis

Adapters were trimmed with cutadapt [27]. Reads were mapped to the transcriptome and unaligned reads to the genome with Spliced Transcripts Alignment to Reference (STAR) version 2.5.2b [28] with the following settings:—outFilterMultimapNmax 20—outFilterMismatchNmax 1—outSAMmultNmax 1. A GTF annotation file accessed from the UCSC genome browser on 11-Sept-2015 was passed to STAR to improve mapping accuracy. Relative changes in ribosome density were determined using the Bioconductor package edgeR (Empirical Analysis of Gene Expression Data in R) [29,30]. edgeR utilizes a negative binomial distributed model for each gene and sample, scaled by library size and relative abundance per experimental group. An empirical Bayes procedure is applied to shrink dispersions towards a consensus value. Ribosome density was estimated via the application of a generalized linear model to determine the interaction between sequence assay (ribosome profiling versus RNA-seq) and condition (Tm-treated versus untreated) while also taking variation between different independent replicate experiments (performed on three different days) into account, using the formula expression level ~ replicate + condition*type + error. The application of an interaction term is a statistically formalized way of detecting which transcripts are translated with different efficiencies upon Tm treatment, as their level of active translation (ribosome profiling) will respond differently to Tm treatment than their total RNA levels (RNA-seq).

Prior to statistical analysis, ribosome footprint reads were separated based on their position in the 5’UTR, the protein coding ORF of the reference transcript 1 (CDS), or the 3’UTR. We did not correct for mapping a read to the first nucleotide of the protected fragment, which was position -13 compared to the protected A-site. As a consequence, the first 4 protected codons of the CDS are mapped to the 5’UTR. In addition, genes with less than 10 cumualtive reads for half of the available samples were removed. The gene list was further filtered on genes containing at least an average 10 RNA-seq reads and an average of 4 ribo-seq reads for all three replicates. This additional filtering step was applied to account for the poly(A) selection, through which transcripts (such as histones) lacking a poly(A) tail are incorrectly identified as significant. Transcripts with a false discovery rate (FDR) < 1% were considered significantly changed. Reported read counts were normalized by counts per million (CPM).

Identification of translation initiation sites (TIS) in Ht treated samples was performed by a previously published bioinformatics peak calling analysis [26]. ORF coordinates were assigned with Mutalyzer [31]. In this analysis, peaks were defined as having >40% of all coverage in the first position and a minimum total coverage of 20. Candidate peaks were considered only if they were a maximum distance of 500nt up- or downstream of an annotated coding sequence (CDS). The maximum coverage for the subsequent 5 downstream codons cannot be higher than the candidate peak, and the candidate peak must have at least 10% of coverage relative to the highest candidate to be considered. Statistical analysis of TIS switching was performed using the R package lme4 (Linear Mixed-Effect Models using ‘Eigen’ and S4) [32]. The model was fitted as previously described [26]. Briefly, fixed effects were assigned for location of the TIS location, Tm treatment, and the interaction between the two. Counts were weighted by library size. Significance between models with and without Tm treatment was determined via a chi-squared likelihood-ratio test and corrected via Benjamini-Hochberg (FDR) at a threshold of 5%.

For UCSC browser snapshots we visualised the peak at the first nucleotide of the RFP and the sum of all three replicates. For metagene analysis we used the RiboGalaxy webtool [33].

## Results

### Tm induced eIF2 phosphorylation and decreased protein synthesis in erythroblasts

**Fig 1:**
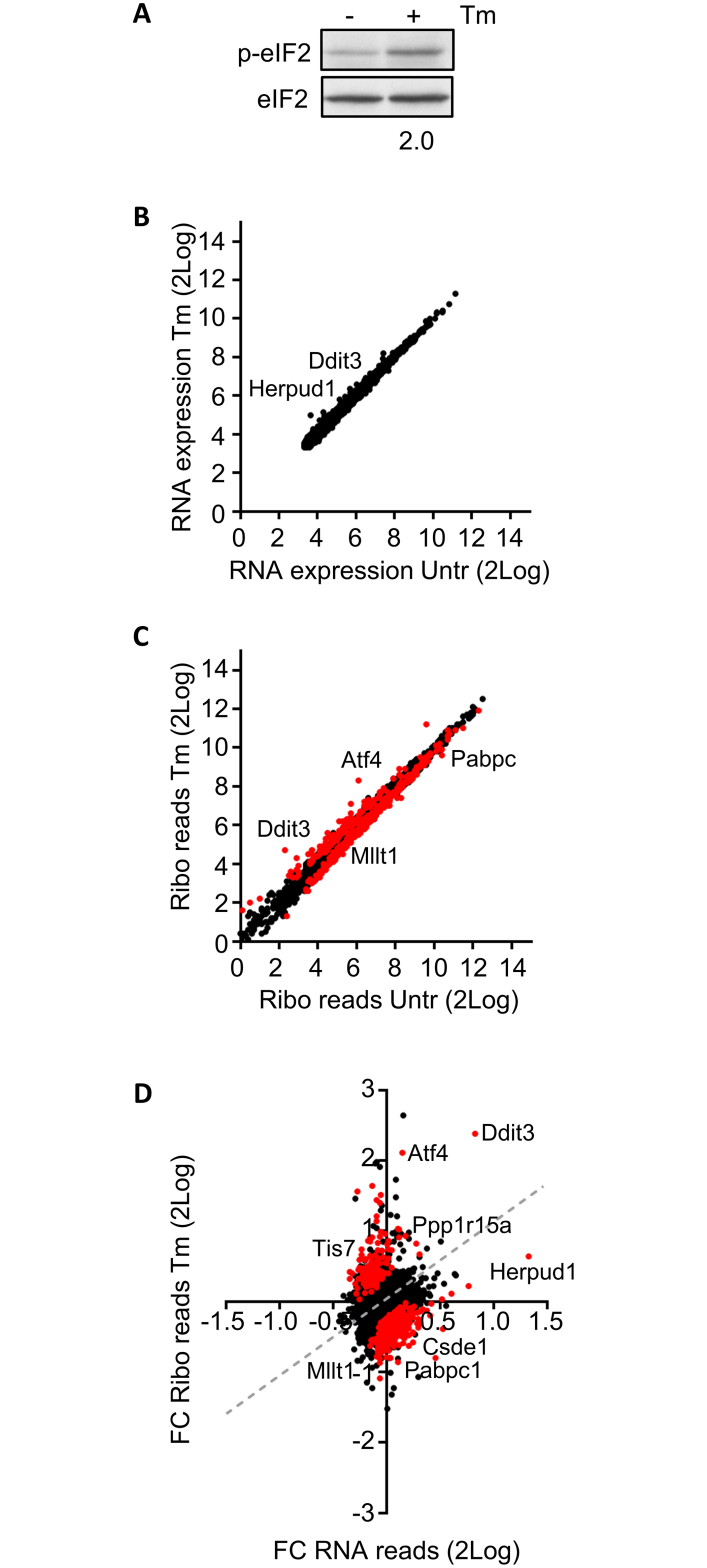
Tm treatment induces phosphorylation of eIF2, reduces protein synthesis and selectively alters ribosome density of some transcripts. (A) Murine erythroblasts (line 15.4) were left untreated (-) or were treated for 90 min with Tm (2.5 μg/ml). Western blots with total cell lysates were probed for phosphorylated (anti P-S51 eIF2) and total eIF2. (B/C) Erythroblast samples as in (A) were processed for polyA+ RNA sequencing (RNAseq; B) or ribosome footprinting (RPF; C). We normalized and averaged sequence reads from 3 biologically independent experiments. For RNAseq We applied a threshold of, on average, 10 reads per condition (B). For RFP reads we applied a threshold of, on average, 1 read per condition (C). A statistical interaction model indicated differential ribosome density on 484 transcripts at a FDR <01%, indicated as red dots. (D) Depiction of 2Log FC ribo reads and RNA reads. Dashed gray line indicates the area where translation follows transcription. The fold-change (FC) in ribo reads (Tm-treated average reads/untreated average reads; Tm/Untr) was plotted against the FC Tm/Untr in RNA expression. Figures are based on data presented in S2 Table.

To evaluate the effect of eIF2 phosphorylation on mRNA translation in erythroblasts we aimed for a rapid induction of eIF2 phosphorylation that minimalizes secondary effects on mRNA expression, stability or translation. Depletion of iron stores is a slow process that takes >4 hours to slow down translation [34]. We treated cells with tunicamycin, which blocks N-glycosylation of proteins and results in ER stress due to an accumulation of unfolded proteins [35]. Average phosphorylation of eIF2 was 2-fold increased upon a 90-minute treatment of erythroblasts with 2.5 μg/ul tunicamycin (Tm) (Fig 1A). Phosphorylation of eIF2 is known to reduce mRNA translation in general [3]. To assess the protein synthesis rate, we measured incorporation of the methionine analogue AHA (L-Azidohomoalanine) in erythroblasts during the 90-minute Tm treatment. Alexa Fluor 488, coupled to AHA, was measured in fixed and permeabilised erythroblasts by flow cytometry. Tm treatment reduced de novo protein synthesis by 35% (Figure A in S1 Fig). To examine whether the reduced protein synthesis rate was due to decreased translation initiation, the polyribosome profile of Tm-treated cells was compared to untreated cells (Figures B-C in S1 Fig). Tm treatment increased the 80S peak and the peak of the first polysome (Figure C in S1 Fig). A shift from heavy towards light polyribosomes and an increase in the 80S monosome peak in Tm-treated cells indicated reduced polysome recruitment. Notably, we did not observe an increase in free ribosomes, rather an accumulation of transcripts with 1 or 2 assembled ribosomes. Together, the results confirmed that Tm treatment of erythroblasts induced eIF2 phosphorylation and reduced mRNA translation.

### Tm-induced changes in mRNA translation

To investigate how eIF2 phosphorylation affects translation of individual transcripts in erythroblasts, we compared the ribosome density of transcripts in absence and presence of Tm. For this, ribosome footprint analysis and mRNA sequencing were performed in parallel on 3 biological replicates harvested on separate days. Following 90 min Tm treatment, cells were treated with 100 μg/ml CHX for 5 min to stall elongating ribosomes. Cells were then harvested for ribosome footprint (RFP) and mRNA sequencing analysis. For RFP analysis the cell lysates were treated with RNase-I, after which the resulting monosomes were purified on sucrose gradients, and RNA was isolated. The rRNA fragments were depleted on beads, the protected fragments were isolated by PAGE, and library preparation was performed as previously described for myoblasts [26]. The number of reads sequenced per replicate was comparable in all replicates (~15 million, S1 Table). We used STAR to map reads to the genome, because of its capacity to correctly map short reads on either side of an intron. On average, 70–80% of reads mapped to genomic locations, 20–30% of reads were too short and therefore discarded. The modal RFP length was 30–32 nucleotides (Figure A in S2 Fig). The presence of two populations with distinct footprint length may reflect the two rotating positions of the ribosome and implies that CHX did not completely stall elongation [36]. Reads were evenly distributed along all chromosomes, which implied that rRNA fragments were efficiently removed (Figure B in S2 Fig). CHX stalls ribosomes, but enables preinitiation complexes to assemble and reach the start codon. CHX-induced accumulation of reads at start codons may be enhanced by Tm [37]. To investigate whether CHX induced an accumulation of reads at start codons we plotted CHX reads 20 nt upstream or downstream of the start codons of the triplicates separately. This indicated that the majority of the protected fragments start at position -13 (frame 3) from the start codon, instead of the commonly observed position -12 (frame 1). Importantly, CHX reads were similarly distributed along the start codon in Tm-treated and untreated cells (Figure C in S2 Fig). These results showed that the combined Tm and CHX treatment did not induce severe side effects during Tm treatment. Metagene analysis of the protected fragments indicated that the majority of the RFPs are in frame 3 (Figure C in S2 Fig). Using the same protocol on myoblasts, we previously found frame 1 as the common frame, which may indicate a change in ribosome composition in erythroblasts that makes it difficult to digest the last nucleotide [26,38]. To use ribosome density as a proxy for protein synthesis from the coding ORF in response to Tm-induced eIF2 phosphorylation, we addressed RFPs in the annotated 5’UTR and the protein coding ORF (CDS) separately. RFPs were mapped to the start of the protected fragment at -13 of the P-site. By consequence, the first 4 codons of the CDS mapped to the 5’UTR and are omitted from the analysis of ribosome density on the CDS.

Tm treatment changes mRNA translation through eIF2 phosphorylation [39], and affects gene transcription through activation of Atf4, Atf6 and Xbp1 [40]. To specifically define the effect on mRNA translation, RFP reads must be corrected for mRNA expression. Aliquots of the same cell samples were processed for polyA+ transcriptome analysis. mRNA reads were normalized (cpm), transcripts with an average read intensity <10 cpm were filtered out. The 2Log transformed mRNA reads derived from Tm-treated and control cells were compared. The short Tm treatment hardly induced changes at the RNA level (Fig 1B, S2 Table), although mRNA expression of some genes, among which *Herpud1* and *Ddit3*, was upregulated by Tm.

Combining RFP and mRNA sequencing allows for a more accurate comparison of ribosome density. We employed a statistical model that examined the relationship between RFP and RNA reads (i.e. ribosome density) for each cell sample and calculated the probability that this relation is similar for Tm-treated and control samples (each in triplicate). At a false discovery rate (FDR) of 1%, Tm treatment increased the ribosome reads in 147 transcripts, and decreased the ribosome reads in 337 (Fig 1C; S2 Table). For these transcripts we calculated the fold change (FC) in RFP and in mRNA reads of Tm-treated over control cells from the average cpm (Fig 1D, S2 Table). As expected, Tm treatment increased the translation of *Atf4* and *Ppp1r15a*, with a limited change in transcription. Tm increased *Ddit3* mRNA expression, but also significantly increased its translation (FC increase in RFP significantly higher than in RNA-seq). Other notable translationally upregulated transcripts were *Ibtk* and *Tis7/Ifrd1*. Among the translationally downregulated transcripts during Tm treatment were *Csde1* and *Dym*. Interestingly, *Herpud1* stands out because its transcription was increased, whereas its translation rate lagged behind (Fig 1D). Top 10 transcripts with increased and decreased ribosome density is shown in Tables 1 and 2, respectively.

**Table 1:** Top 10 transcripts with increased ribosome density and their function (see also S2 Table).

Upregulated transcripts				
Name	Full name	Function	FDR	2Log FC
Atf4	Activating transcription factor 4	Transcription factor; apoptosis	1.09E-36	1.96
Scoc	Short coiled-coil protein	Autophagy	7.83E-11	1.46
Ibtk	Inhibitor of Bruton tyrosine kinase	Downregulates BTK kinase activity; apoptosis	1.80E-10	1.04
Pnrc2	Proline Rich Nuclear Receptor Coactivator 2	Nonsense-mediated mRNA decay	2.14E-08	1.04
Ddit3	DNA Damage Inducible Transcript 3	Transcription factor; apoptosis	2.30E-08	1.55
Ppp1r15a	Protein Phosphatase 1 Regulatory Subunit 15A	PPase1 subunit, involved in dephosphorylation of eIF2	4.93E-08	0.92
Ost4	Oligosaccharyltransferase Complex Subunit 4	Post-translational modification	8.12E-08	0.79
Usmg5	Up-Regulated During Skeletal Muscle Growth 5 Homolog	Mitochondrial Role	1.18E-07	1.51
Gtf2h5	General Transcription Factor IIH Subunit 5	DNA repair Process	1.64E-07	0.61
Dpm2	Dolichyl-Phosphate Mannosyltransferase Subunit 2	Post-translational modification	2.09E-07	0.80

**Table 2:** Top 10 transcripts with decreased ribosome density and their function (see also S2 Table).

Downregulated transcripts				
Name	Full name	Function	FDR	2Log FC
Hspa5	Heat Shock Protein Family A (Hsp70) Member 5	ER membrane transport	3.20E-17	-0.55
Hbs1l	HBS1 Like Translational GTPase	Translation	4.50E-10	-0.55
Anapc1	Anaphase Promoting Complex Subunit 1	Ubiquitination	1.55E-09	-0.46
Tnks2	Tankyrase 2	Ubiquitination	2.54E-09	-0.55
Pcsk6	Proprotein Convertase Subtilisin/Kexin Type 6	Protease	7.62E-08	-0.67
Cep192	Centrosomal Protein 192	Cytoskeleton	1.29E-07	-0.48
Rbm17	RNA Binding Motif Protein 17	RNA-binding protein; mRNA splicing	1.37E-07	-0.49
Efr3a	EFR3 homolog A	G protein-coupled receptor phosphorylation	1.84E-07	-0.65
Hsd17b12	Hydroxysteroid 17-Beta Dehydrogenase 12	Lipid metabolism	3.22E-07	-0.43
Csde1	Cold Shock Domain Containing E1	RNA-binding protein; mRNA splicing	3.99E-07	-0.83

### Pathways that were affected by the Tm treatment

We investigated which pathways were altered by transcripts with significantly altered ribosome density using overrepresentation analysis (ORA) with Genetrail2 [41]. Increased ribosome density was foremost associated with transcripts encoding proteins of mitochondria, mitochondrial and endoplasmic reticulum components (enrichment p<10^−6^), followed by transcription complex (p = 1.6x10^-3^) (S3 Table). [42]. Among molecular processes, transcriptional (co)activator complexes were most enriched (p = 1.3x10^-4^). The ISR response factors Atf4 and Ddit3 directly bind DNA to induce transcripts involved in cell survival or apoptosis [40]. The transcription factors Gtf2h, Mbd3, JunB and Kmt2e, were also enriched among transcripts with increased ribosome density. For transcripts with more than average decreased ribosome density, the top 30 pathways are shown in S4 Table, according to the adjusted p-value. Among molecular mechanisms, the most enriched transcripts were associated with kinases, and control of kinase activity (p<10^−10^). The second most enriched, and independent molecular function was again transcription activation and chromatin (p = 10^−9^). In conclusion, prolonged phosphorylation of eIF2 will reprogram erythroblasts through altered expression of multiple transcription factors, which may stabilise a “stress phenotype” of erythroblasts.

### Detection of translation start sites

In parallel with the CHX treatment, cells were treated with 2μg/ml Harringtonine (Ht) for 7 min to stall initiating ribosomes at start codons, while associated ribosomes complete translation and run off the transcripts. Following quality control, we obtained 11 to 15 million reads per individual sample (triplicate experiments with and without Tm) of which an average of 60% could be mapped to the genome using STAR (S1 Table). We combined STAR with a previously described script that maps the first nucleotide of the RFP and predicts the corresponding translated codon [26]. Similar to the CHX-stabilised RFPs, also the Ht-stalled RFPs mainly started in frame 3 (Figure A in S3 Fig). Accordingly, most protected reads started at position -13 relative to the annotated start codon (Figure B in S3 Fig). Because test runs already showed the preferential protection of 13 nt, we had increased the RNAse-I concentration compared to the original protocol that yielded reads starting in >80% at the -12 nucleotide position [26]. This did not make a difference in the length of the pattern of protected fragments. We separated protected fragments according to read length, but longer and smaller fragments were similarly distributed over -12 and -13 (Figure B in S3 Fig). Therefore, in our TIS peak detection, we called peaks at both positions.

The cumulative reads of the triplicate for each condition, were entered into the previously developed peak calling algorithm to identify translation initiation sites (TIS) [26]. Based on their position in the consensus transcript, peaks were segregated to TISs in 5’UTR, annotated start codon, in the CDS, or in the 3’UTR. Peaks were assigned to AUG, CUG, GUG or UUG start codons at either +12 or +13 from the start of the RFP. All other peaks were assigned to the codon at the +13 position counted from the top of the peak. A total of 1940 and 2175 TISs were identified in the annotated 5’UTRs of transcripts in untreated and Tm treated cells, respectively (S5 Table). These corresponded to corresponded to 1467 and 1666 genes, respectively because several uORFs can be translated (Figure C in S3 Fig). The CDS of untreated and Tm-treated cells revealed 1935 and 2045 TISs, respectively. We observed a preference for CUGs in the 5’UTR, and for AUGs in the CDS, similar to what has been reported (S4 Fig, S6 Table) [21,26]. Overall 53 and 47% of TISs in the 5’UTR were [A/C/G/U]UG startcodons, but these codons only comprised 23 and 26% of all TISs in the CDS. TIS that did not represent [A/C/G/U]UG startcodons were not randomly distributed over codons, but mainly mapped to triplets encoding the large, and positively charged amino acids Arginine (R) and Lysine (K) (24% in the 5’UTR, 28 and 30% in the CDS; S4 Fig). The TISs at R and K codons are only present upon Ht treatment, not upon CHX treatment (S5 Fig, example in *Abce1*). This suggested that these TIS on R and K codons are Ht artefacts and not ribosome pausing sites.

Therefore, we only considered [A/C/G/U]UG start codons as real TISs in the 5´UTR as well as in the CDS. As a result, detection of TISs was limited to 867 transcripts in untreated erythroblasts and in 907 transcripts in Tm-treated erythroblasts. In most transcripts we detected 1 TIS. The maximum number of detected TISs in the 5´UTR was 4 in the case of *Eri3* (*Exoribonuclease Family Member 3*) (Figure C in S3 Fig, S5 Table). Taken together, however, uORF translation is widespread among expressed genes in both conditions.

### Control of mRNA translation is poorly predicted by uORFs

In theory, comparison of TIS peak intensities corresponding to annotated start codons should validate the differences in ribosome density. Increased or reduced ribosome density should be mirrored by increased or reduced peak height on the start codon. However, start sites hardly accumulate reads when they are located downstream of an uORF, and the division of the peak over the -12 and -13 position also complicated quantitative analysis. The analysis of ribosome density was much more accurate than an analysis of peaks on annotated start sites. Therefore, we focussed on the presence of unexpected start sites within the CDS that may give rise to proteins with distinct N-termini. We considered all genes with at least 1 observed [A/C/G/U]UG consensus start codon TIS in the 5’ UTR. For 683 genes we identified consensus start codon TISs under both control and Tm-treated conditions. The high overlap (79% of the lowest number) is expected, because the first TIS peak accumulates during Ht treatment while the formation of pre-initiation scanning complexes and scanning from the cap continues. In these 683 transcripts we detected a TIS in the CDS of 41 transcripts: 21 TISs in the CDS of transcripts of both TM-treated and untreated condition, 12 TISs only in the transcripts of Tm-treated cells, and 8 TISs only in transcripts of control cells. A detected peak in the coding sequence may indicate translation of an ORF that leads to a protein isoform. An example is *Transcription factor cp2* (*Tfcp2*) which is translated from the annotated start codon embedded in a strong Kozak consensus sequence. A second very strong TIS peak maps downstream of the start codon in the CDS. However, it does not correspond to a N-terminally truncated protein but to a 9-codon small ORF (S6 Fig), which appeared to be the case for more peaks in the CDS. Therefore, we assessed which TISs actually lead to significant differential translation of protein isoforms during Tm exposure. To do this, we investigated whether Tm treatment changed the peak intensity ratio between TIS peaks within a transcript as previously described [26]. The ratio between triplicate TIS peak reads at distinct positions within a gene was compared between untreated and Tm-treated cells. At a p-value less than 0.01, few transcripts revealed differentially employed TISs in their 5’UTR (S7 Table). For example, the ratio between the TIS detected in the 5’UTR of *Ranbp1* and the TIS of the annotated CDS start codon differed significantly dependent on Tm treatment (S7 Fig). Interestingly, *Ranbp1* RNA expression in erythroid progenitors is high compared to CD34+ cells [43]. In conclusion, we did not detect major changes in the expression of protein isoforms upon phosphorylation of eIF2.

Next, we investigated the role of uORFs in the quantitative control of RNA translation. We hypothesized that uORFs render translation of the protein coding ORF more sensitive to eIF2 phosphorylation. To assess whether increased, or decreased ribosome density in the CDS upon eIF2 phosphorylation is due to uORF translation, we considered transcripts with at least 1 detected TIS peak in the 5’UTR, in presence and absence of Tm. Within this subset we compared transcripts characterised by significantly up- or downregulated ribosome density (FDR ≤ 0.05) (Fig 1E, S2 Table) with control transcripts that were not specifically affected by eIF2 phosphorylation (FDR>0.5 for differential RFP density). Surprisingly, the distribution of transcripts with or without TISs in the 5´UTR was the same for all transcripts independent of Tm-dependent ribosome density (Table 3; Pearson’s Chi-square, p-value not significant). These results suggest that translation of an uORF may not be a strong predictor of either quantitative or qualitative mRNA translation.

**Table 3:** Distribution of TIS peaks in significantly altered transcripts and unaffected (control) transcripts with FDR ≤ 0.05.

TIS Peak calling		
% genes	TIS Peak	No TIS peak
Up	19	82
Down	22	77
Control	22	78

### Long uORFs with a CUG start codon occur commonly in transcripts with Tm-enhanced translation

**Fig 2:**
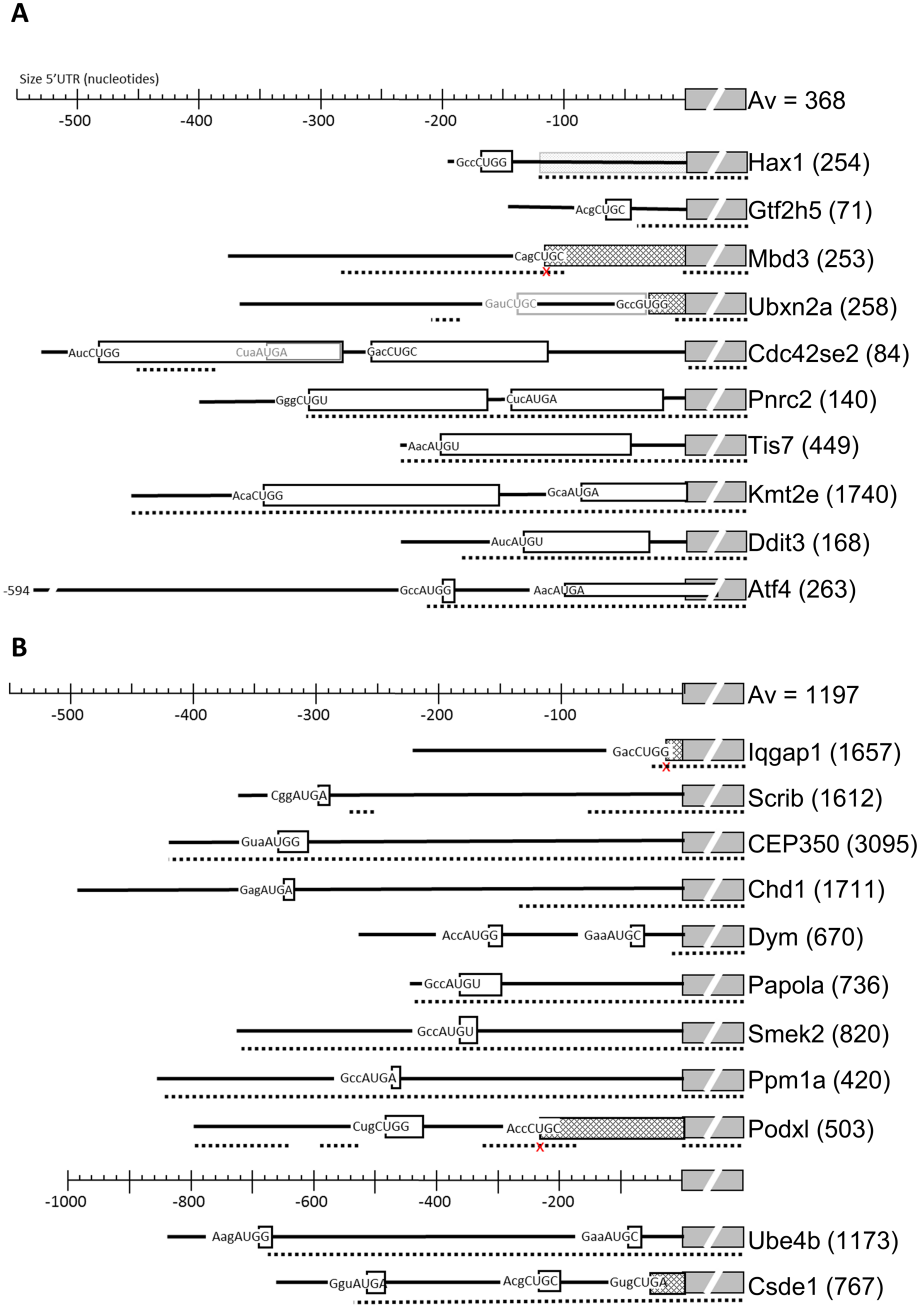
Position and length of uORFs in the 5´UTR of transcripts subject to Tm-controlled translation. (A, B) Top line indicates the distance in nt upstream of the main annotated start codon. The same relative size is used for transcripts with Tm-enhanced translation (A), or Tm-decreased translation (B), except for the last two transcripts for which size was condensed 2-fold as indicated by a separate size marker. The collapsed annotated protein ORF is shown by a grey interrupted box, with the protein name directly at the rightside. Numbers between brackets indicate the size of the main annotated protein in amino acids. Adjacent boxes with a fence pattern on the left of the “protein box” indicate a N-terminal extension of the protein. A fenced box at the back ground as shown for Hax1 indicates that this part of the annotated protein seems not translated. All uORF are indicated by open boxes, and the start codon is written at the start of the box including its Kozak context. The dashed line below indicates areas that are >90% conserved between mouse and man. A small cross below the start codon in a conserved area indicates that the start codon is not conserved. Conserved areas were identified by Blastn with the mouse sequence on the human transcriptome.

For individual transcripts, the translation of uORFs can be crucial for proper regulation. For transcripts on which ribosome density was up- or more than average downregulated in response to Tm treatment, we established the TIS positions (Ht-induced TIS peaks) and the sizes of corresponding uORFs (RFPs protected in presence of CHX) (Fig 2A and 2B). We first analysed 10 transcripts with Tm-increased ribosome density and upstream TISs. We detected 14 TISs in the 5’UTR of these 10 transcripts: 2 UGU, 6 CUG and 6 AUG codons. From the 6 AUG codons 4 mapped to the known targets *Atf4*, *Ddit3* and *Tis7*. Thus, the novel, experimentally determined TISs were mainly non-AUG. These non-AUG TISs that we established experimentally are hard to predict, particularly when they occur in a poor Kozak consensus sequence (e.g. the Cag CUG C start codon in *Mbd3*).

**Fig 3:**
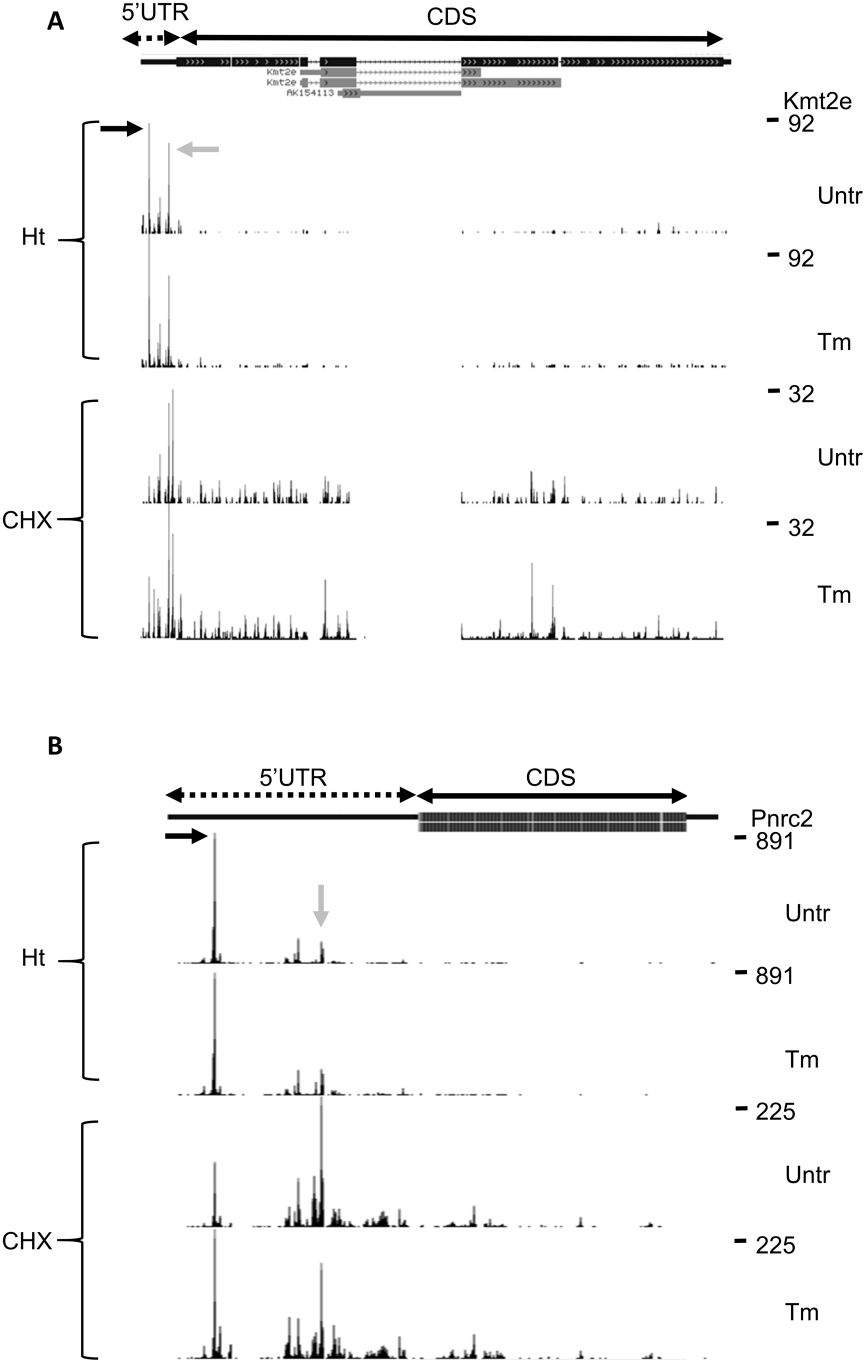
Web browser snapshot of the 5´UTR of *Lysine methyltransferase 2E (Kmt2e)* and *Proline Rich Receptor Coactivator 2 (Pnrc2)*. (A/B) Aggregate Ht- and CHX-stabilized RFP counts from three replicates of Tm-treated and untreated (Unt) cells are mapped to the genome and visualized in the UCSC web browser. Numbers on the right-hand side indicate maximum read counts in the respective lane. Only the start of the protein coding ORF is shown. (A) The data indicate two uORFs on the 5’UTR of *Kmt2e*. Black and gray arrow indicates Ht and CHX peak that maps to the start codon uORFs 1 and 2, respectively. uORF2 overlaps the start codon of *Kmt2e*. (B) The data indicate two uORFs. Black arrow indicates a TIS at start codon for uORF1, gray arrow indicates a TIS for uORF2.

The mechanism employed by *Atf4*, a small uORF followed by an inhibitory uORF overlapping the protein codon TIS, appeared unique for *Atf4*. In only two other transcripts small uORFs were translated (*Hax1* and *Gtf2h5)*, and in only one transcript a second uORF overlapping the protein start codon was translated (*Kmt2e*) (Fig 3A, gray arrow), leading to unaffected CDS translation during Tm treatment, as shown by Ht and CHX peaks. Strikingly, the annotated start codon of *Hax1* was skipped, and an AUG codon 120 nt downstream was used as the TIS for the *Hax1* coding frame. The GWIPS website (http://gwips.ucc.ie/) [44] revealed that this occurs in most mouse cells. The novel TISs in *Mbd3* and *Ubxn2a* appeared to be in frame with the known CDS and initiated an N-terminally extended protein isoform. Comparison with global data on the GWIPS website indicated that this is common for *Mbd3* in mouse cells. In contrast, most cell types are protected from the extension of *Ubxn2a* by a large uORF that ends just 1 codon upstream of the TIS. This uORF was hardly expressed in erythroblasts according to both Ht- and CHX-induced RFPs. The N-terminally extended isoforms of *Mbd3* and *Ubxn2a* are not conserved between mouse and human.

In five transcripts one or two long uORFs were translated, four of these are >90% conserved between mouse and human. These uORFs are also translated in other celltypes (GWIPS data), although at different ratios. Strikingly, an AUG codon within the first long uORF of *Cdc42se2* is the major TIS detected in most other cells. In our data this was a minor start, and we found a major contribution of the two long uORFs, both in Ht- and in CHX-arrested RFPs. *Pnrc2* encodes a transcriptional co-activator of the glucocorticoid receptor and Tm treatment induced a 1.9-fold increase in *Pnrc2* ribosome density. Two large uORFs that are located close to each other are both translated as can be inferred from the CHX reads. The CDS, however, is poorly translated. Tm treatment resulted in a shift between the occupancy of the uORF start codons with more reads on the 1^st^ start codon and less on the 2^nd^ start codon (Fig 3B), which may create more space for reinitiation and CDS translation.

### A long 5’UTR with a short uORF harbouring an AUG TIS is common in transcripts undergoing Tm-reduced translation

**Fig 4:**
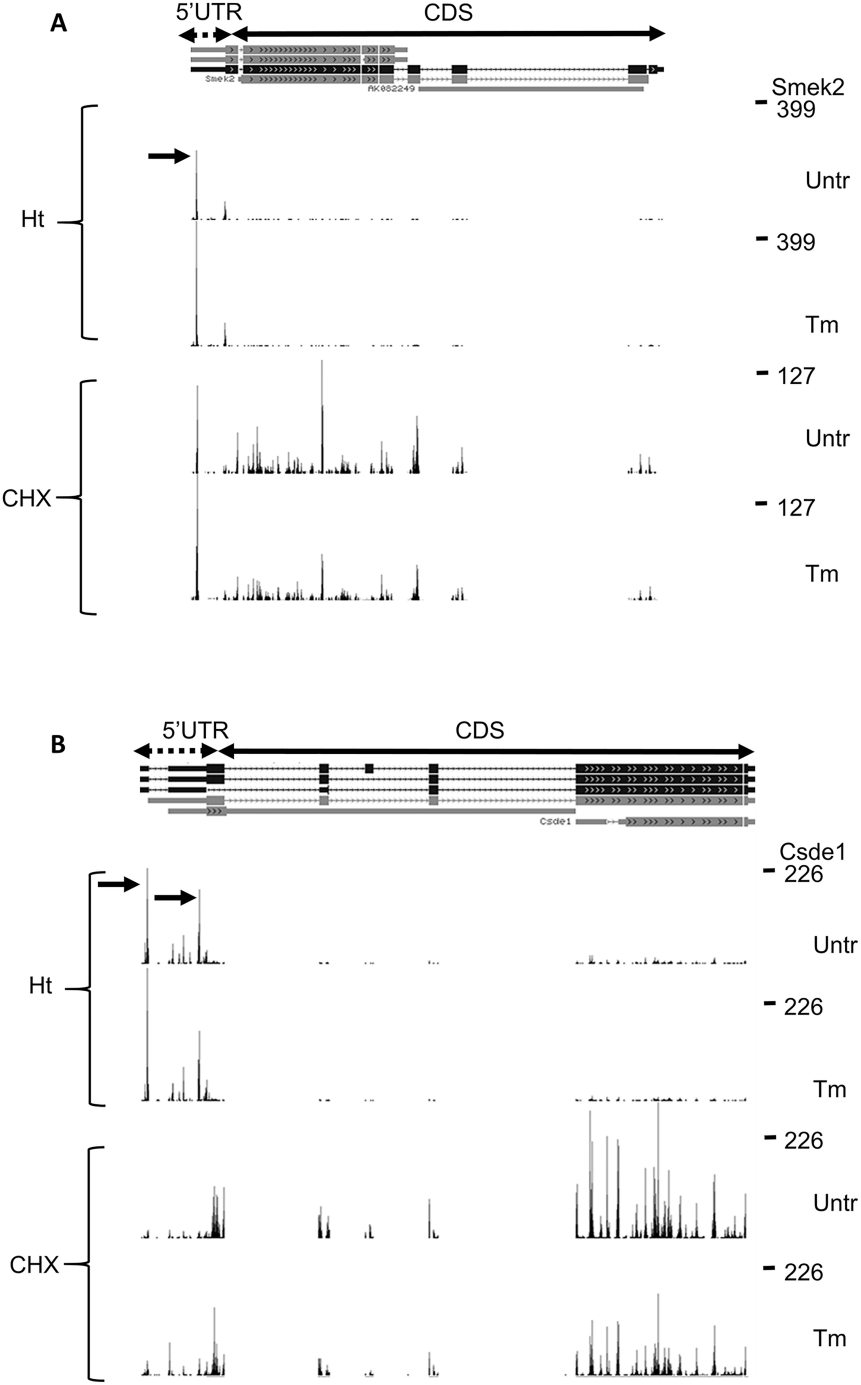
Web browser snapshot of the 5´UTR of *protein phosphatase 4 regulatory subunit 3B (Smek2)* and *Cold shock domain containing E1 (Csde1)*. (A/B) Aggregate Ht- and CHX-stabilized RFP counts from three replicates of untreated (Untr) and Tm-treated cells were mapped to the genome and visualized in the UCSC web browser. Numbers on the right-hand side indicate maximum read counts in the respective lane. Only the start of the protein coding ORF is shown. (A) The data indicate a small uORF on the 5’UTR of *Smek2* (gray box). Horizontal arrow indicates Ht and CHX peak that maps to the start codon of this uORF (B) The data indicate two uORFs that are depicted by grey boxes. Gray arrows indicate TISs at uORF start codons, fenced box indicates N-terminal extension and black arrow indicates the TIS of this extended protein.

The start codon, length, and position of uORFs in transcripts with more than average Tm-decreased translation was different from the uORFs found in upregulated transcripts (Fig 2A and 2B). Whereas we detected many long uORFs in transcripts with Tm-enhanced translation, all uORFs detected in transcripts with Tm-reduced translation are short. In 11 transcripts (>2-fold reduction in ribosome density compared to average) we observed 15 TISs, 11 of which were AUG codons. For example, *Smek2* has a short single uORF, that is highly translated, with clear Ht and CHX peaks that shows increased translation during Tm, which leads to a reduction of CHX reads in the CDS (Fig 4A, black arrow). For 3 of the 11 transcripts we observed an N-terminal extension (*Csde1*, *Iqgap1*, *Podxl*), that are also observed in other cell types but at a lower frequency (GWIPS comparison). In addition, we observed that reads mapped to two small uORFs in the 5’UTR of *Csde1* (black arrows), it has to be noted however that according to the CHX reads these uORFs are not highly translated, compared to the CDS (Fig 4B). The uORF of *Chd1* is not detected in other cell types, whereas an additional, further upstream, uORF was detected for *Ppm1a* and *Csde1* in many other cell types, but not in our erythroblasts (GWIPS comparison). The 5’UTR of seven transcripts is >90% conserved between mouse and man, suggesting conserved mechanism of translation control. Notably, 9/10 transcripts subject to Tm-enhanced translation encoded short proteins (average of all encoded proteins is 368 amino acids). In contrast, the average of protein size encoded by transcripts subject to Tm-decreased translation is 1197 amino acids.

## Discussion

Iron deficiency, oxidative stress, or the presence of unfolded proteins in erythroblasts activates the eIF2 kinases HRI and PERK, respectively, which results in phosphorylation, and thereby inactivation, of eIF2. This decreases overall mRNA translation to prevent for instance the accumulation and aggregation of globin polypeptides in absence of iron and heme [5]. To characterise the molecular pathways and cellular processes that respond to eIF2 phosphorylation in erythroblasts we combined ribosome profiling and transcriptome analysis to detect transcripts with increased ribosome density, or with a more than average decreased density of elongating ribosomes upon eIF2 phosphorylation. We found, among others, known components of the ISR pathway to be increased in translation, such as *Atf4*, *Ddit3*, and *Tis7*, but also transcripts that are less well known to be translated upon eIF2 phosphorylation including *Prnc2*, that encodes a protein involved in recruitment of transcripts to P-bodies for subsequent degradation [45]. On the other hand, Tm treatment also led to more than average downregulation of translation for a set of transcripts that included *Csde1* and *Dym*. Whereas stabilisation with CHX identified elongating footprints, the treatment of erythroblasts with Ht identified footprints at translation initiation sites. Combination of CHX and Ht RFPs showed that the presence of a translated uORF did not predict the sensitivity of a particular mRNA during eIF2 phosphorylation. The high degree of conservation between the 5´UTR of man and mouse suggests that the translation mechanism may be more complex than only the presence of uORFs. Strikingly, transcripts with Tm-enhanced translation contained long, conserved uORFs that often started with a CUG start codon, whereas transcripts with Tm-reduced translation contained short uORFs starting from an AUG codon. Because CHX and Ht RFP reads do not quantitatively represent ribosome density at start sites, which is a dominant contribution also for the CHX reads on a short uORF, it is important to validate the roles of the uORFs in translational control of these transcripts.

Some of the transcripts that we found to be translationally upregulated upon Tm treatment of erythroblasts were recently linked to eIF2 phosphorylation in HEK293 cells. These transcripts encoded proteins involved in the ISR such as Atf4, Atf5, and Ppp1R15a/Gadd34, Ibtk, and Tis7 [9,46]. The ISR is highly conserved between eukaryotes, from yeast to mammals [47]. Several ribosome profiling datasets were published that address the ISR, but the data are difficult to compare. Moreover, these studies do not address initiating ribosomes. Lack of uniformity in methods, in induction of eIF2 phosphorylation, in statistical analysis and in cell types complicates comparisons between these studies. Nevertheless, we compared the transcripts with increased translation in erythroblasts to transcripts with increased ribosome density in response to arsenite treatment of HEK293 cell [9]. The reason to use this database is due to the short interval of Tm treatment, in which translational changes are observed due to eIF2 phosphorylation, similar to the setup in this study. Whereas we (*i*) identified differential ribosome density in erythroblasts, and (*ii*) used a statistical interaction model to compare RFP and RNAseq reads. Andreev et al. (*i*) calculated translation efficiency in HEK293 cells, and (*ii*) determined the Z-score for the fold-change in translation efficiency. They considered transcripts with a Z-score>4 as significantly upregulated. For this comparison we considered the transcripts with a Z-score>3 in the dataset of Andreev et al. (S8 Fig). Strikingly, the overlap between differentially translated transcripts was limited to *Atf4*, *Atf5*, *Ppp1R15a*, *Slc35A4* and *Tis7*. There was a clear separation between transcripts that were differentially translated in HEK293 cells or in erythroblasts. The level of eIF2 phosphorylation (and thereby the amount of available eIF2) was different in Andreev et al. compared to our study. Perhaps varying amounts of available eIF2 leads to altered translational control of some mRNAs. However, this difference may also reflect an essential difference between these two cell types. We hypothesize that the ISR downstream of eIF2 phosphorylation is different in erythroblasts compared to HEK293 cells. The activity and specificity of eIF2 is modulated by the association with eIF1 and eIF5 [48]. eIF1 is upregulated in response to SCF-induced erythroblast expansion, whereas eIF5 is upregulated during differentiation to hemoglobinised, enucleated red blood cells [18]. Interestingly, cancer cells were also shown to modify their response to eIF2 phosphorylation by expression of the alternative translation initiation factor eIF2A [49]. The effect of eIF2A only becomes apparent when eIF2 is limiting [50]. Thus, depending on the expression levels of various translation initiation factors, each cell may respond differently to eIF2 posphorylation, because translation of uORFs and protein coding ORFs will depend on the combination of eIF2 availability plus the modulation of its activity and specificity by associated initiation factors.

Among differentially translated transcripts we did not observe transcripts with erythroid specific expression. Nevertheless, Csde1 is strongly upregulated in erythroblasts and we previously reported reduced Csde1 expression in Diamond Blackfan Anemia [19]. Ago2 and Dicer have a very general role in miRNA-mediated mRNA degradation, but loss of Ago2 specifically affects erythropoiesis [51]. We did not detect erythroid specific transcripts among the transcripts with a Tm-induced increase in ribosome density, but there is ample evidence that the ISR is crucial for erythropoiesis[52].

Interpretation of RFP data sets, and particularly of translation initiation sites is complicated by several factors including (*i*) sequence depth, (*ii*) ligation bias, and (*iii*) TIS peak imbalance. First, each read is a single count on a single codon. A substantial number of reads need to map to each codon position to identify changes in codon usage that are statistically significant. From samples treated with CHX we obtained a total of >45 million reads for the combined triplicate. Statistical analysis uses the individual experiments. Thus, peaks that can be discerned in the UCSC web browser may still lack statistical power. Second, we observed that ligation of the small RFP fragments to adapter oligonucleotides is very sensitive to bias and that this bias depends on the ligation kit. We detected the start codon of the first uORF of *Atf*4 in pilot experiments, but the final experiment only showed a relatively low number of reads at this position. We cannot exclude the possibility that the use of a different adapter ligation kit introduced bias in the ligation step. In agreement with this supposition, ribo-seq profiles of *Atf*4 also show a loss of uORF1 in other studies that used the same library prep kit [53,54] compared to studies that use different methods, as shown in the GWIPS-viz genome browser [44]. Third, the detection of TISs following Ht treatment has a strong bias towards the most upstream uORF. Ht or CHX do not inhibit the association of the pre-initiation scanning complex at the cap, and scanning to the first start codon. During treatment, this first peak continues to grow, while all other peaks downstream of the first peak depend on scanning complexes present between the peaks at the start of the treatment.

Finally, we also observed an enrichment of Ht peaks at codons that code for Arginine (R) and Lysine (K). These amino acids are positively charged, and they are among the bulkiest amino acids. The triplets coding for other bulky amino acids (tyrosine, Y; Phenylalanine, F) are not enriched among the peaks. Having a positively charged (large) amino acid at the P-site of the ribosome may either create more space at the A-site to bind Ht, or it may pause ribosome progression. In the latter case ribosome density should also be increased upon CHX treatment. Therefore, TIS peaks are subject to bias and need to be interpreted with caution. In combination with elongating RFPs, however, it is a powerful method to identify uORFs. Ribosome profiling on other cell types reported different biases [21,55]. This may be due to technical details such as bias in the isolation and ligation of protected fragments, but it could also hint at a cell type specific composition of the pre-initiation scanning complex and elongating ribosomes.

The data also show that many alternative start codons, particularly CUG, are used as TISs. Therefore, prediction of uORF translation from the primary transcript sequence is difficult, if not impossible. Experimental TIS analysis such as the Ht treatment to stall ribosomes at start codons, is needed to understand how TIS may contribute to control translation in specific transcripts. Selective translational control by eIF2 is performed through differential start codon recognition and the presence of uORFs on 5’ UTRs of specific mRNAs [4]. However, in our proteotoxic stress model we did not find an enrichment of uORF containing transcripts. The translation of uORFs appeared widespread.

The transcripts with significantly altered translation compared to the average change in translation caused by Tm were enriched for CDS giving rise to transcription factors, like Pnrc2, Tis7, Kmt2e and JunB. Pnrc2 interacts with the glucocorticoid receptor to induce mRNA decay of some transcripts [56]. Glucocorticoids are important for expansion of the erythroblast compartment upon induction of stress erythropoieses [57]. Interestingly, JunB was reported to drive erythroid differentiation [58]. Increased expression of JunB in response to eIF2 phosphorylation may be a convergence node in erythropoiesis for ER-stress and activation of stress kinases of the MAPkinase pathway similar to what was found for pancreatic cells [59]. Tis7 was found to be upregulated in chicken erythroid cells during hypoxic stress [60]. Kmt2e regulates cell cycle progression in myoblasts [61]. These transcription factors could also be involved in activating the transcription of other proteotoxic stress responsive genes and induce a cell survival mechanism in erythroblasts.

In conclusion, translational control by eIF2 in erythroid cells is important for maintaining red blood cell function and survival. In this study we have used ribosome profiling to investigate which transcripts are translationally up or downregulated during ER stress in erythroblasts. Unexpectedly, uORFs are not enriched in these transcripts. We also observed [A/C/G/U]UG TISs within the CDS of 179 transcripts, and these were mostly short out-of-frame ORFs. Whether these are unimportant side effects due to leaky scanning of the CDS starting codon, whether their translation interferes with the translation of the CDS, or whether the encoded peptides are stable is not known and needs to be investigated. Future studies should be performed to gain more insight into control of translation by eIF2, and to understand the role of these encoded proteins in erythropoiesis.

### Accession numbers

Original sequencing results have been deposited in the BioProject Database under project ID PRJNA380970.

### Data access

UCSC browser session:

https://genome.ucsc.edu/cgi-bin/hgTracks?hgS_doOtherUser=submit&hgS_otherUserName=ksm113&hgS_otherUserSessionName=TIS%20Ifrd1%20Har%20%26%20Chx

SubmissionID: SUB2489513

BioProject ID: PRJNA380970

BioSample accessions: SAMN06660139, SAMN06660140

http://www.ncbi.nlm.nih.gov/biosample/6660139

http://www.ncbi.nlm.nih.gov/biosample/6660140

## Supporting information

S1 Fig Tm treatment causes a reduction of translation. (A) Protein synthesis was measured by Click-it technology. Incorporated methionine analogue AHA was coupled to Alexa Fluor 488, and measured by flow cytometry (BD LSR-II). (average values, n = 3, for every pair untreated cells were set to 1, error bar indicated StDev, star indicates p<0.05). (B-C) Cell lysate was density separated on a 17–50% sucrose gradients and the absorbance at 254nm was measured throughout the gradient, which is a measure for RNA. The polysome profile of untreated cells (B) shows large polysomes with a relatively small monosome peak, whereas Tm-treated cells displayed an accumulation of light polyribosomes (representative plots from 3 independent experiments) (D) Quantification of the area under the curve (n = 3).
(TIF)

S2 Fig Ribosome profiling data quality. (A) Ribosomes were stabilised with CHX. Shown is the fitted line through the average values of three biological replicates harvested following Tm treatment or three control replicates. Error bars indicate standard deviation. (B) RFP fragments were mapped to the genome and the number of reads (all experiments combined) was enumerated per chromosome. Shown is the percentage of all reads mapping to the different chromosomes. (C) RFP sequence data were uploaded to the RiboGalaxy webtool. The start of each RFP was mapped to the genome. The number of reads starting at position -20 to +50 compared to the startcodon, and on position -50 to +20 compared to the stopcodon were calculated for reads of 32 nt. Reads in each frame are indicated by distinct colors. Red: frame 1, green: frame 2, blue: frame 3. Representative plots of one replicate of each condition is shown.
(TIF)

S3 Fig Harringtonine-induced RFP are mostly translated in frame 3. We used STAR to map Ht-stabilized RFP to the genome, and used our previously described script to map the first nucleotide relative to the annotated reading frame. Shades of blue (a2, b2, c2) represent RFP from untreated cells, shades of orange (a4, b4, c4) represent RFP from Tm-treated cells. (B) The start of the protected RFP fragment, was mapped relative to the the annotated start codon. The start codon is located on position 0, 1, 2 and represents the P-site of the ribosome (because Ht blocks the E-site). The number of RFP reads starting at each position relative to the start codon is indicated. **(C)** The number of Ht peaks (potential TIS) that were detected in the annotated 5’UTR of individual genes (U: no TIS detected).
(TIF)

S4 Fig Harringtonine preferentially stalls ribosomes at R and K codons. Mapped Ht RFPs were analysed with a peak calling program to define potential TISs in the 5’UTR (top) or CDS (bottom) in cells treated with Tm (right side) or untreated (left side). In the 5’UTR almost half of the detected TIS represented canonical (AUG) and noncanonical (CUG, UUG, GUG) startcodons, whereas only ~25% of all peaks in the CDS represented canonical or noncanonical start codons. The amino acid (1 letter) code of non-start codons was added to the codons that were most frequently detected as putative TIS. Exact percentages and codons are presented in supplemental S6 Table.
(TIF)

S5 Fig Web browser snapshot of *ATP-binding cassette sub-family E member 1* (*Abce1)*. Cumulative Ht- and CHX-stabilized RFP counts from Tm-treated and untreated (Unt) cells are mapped to the genome and visualized in the UCSC web browser. Numbers on the right-hand side indicate maximum read counts in the respective lane. Gray lines indicate introns. The arrow indicates a peak of Ht-stabilised RFP that corresponds to a non-start codon. This peak is not present in CHX-stabilised RFP, indicating that this is most likely a Ht-induced artefact.
(TIF)

S6 Fig Web browser snapshot of *Tfcp2*. Aggregate Ht- and CHX-stabilized RFP reads from Tm-treated and untreated (Unt) cells are mapped to the genome and visualized in the UCSC web browser. Numbers on the right-hand side indicate maximum read counts in the respective lane. Arrows indicate Ht peaks. Gray lines indicate introns. Part of the 3’UTR is cropped.
(TIF)

S7 Fig Web browser snapshot of *Ranbp1*. Cumulative Ht- and CHX-stabilized RFP counts from Tm-treated and untreated (Unt) cells are mapped to the genome and visualized in the UCSC web browser. Numbers on the right-hand side indicate maximum read counts in the respective lane. Gray lines indicate introns. The uORFs in the 5’UTR and the protein coding ORF (CDS) are indicated.
(TIF)

S8 Fig Comparison of ribosome occupancy in response to eIF2 phosphorylation in HEK293 cells (Andreev et al.) and mouse erythroblasts (this study). Triangles indicate transcripts of which translation is similarly upregulated upon eIF2 phosphorylation in both studies. White circles represent transcripts with enhanced translation (Z-score >3) in HEK293 cells but not in mouse erythroblasts; dark grey circles represent transcripts with enhanced translation in mouse erythroblasts (FDR<0.01) but not in HEK293.
(TIF)

S1 Table Overview of ribosome footprint reads mapped with STAR. Ribosome reads were mapped with STAR to the genome. This table gives an overview of read length and how many reads mapped to the genome for each sample. Note: Multi-mapped reads were not discarded, unless they mapped to more than 20 locations.
(XLSX)

S2 Table Normalised sequence counts for ribosome footprints (RFP) and pA+ RNA sequencing (counts per million; cpm). 2Log normalized RFP reads (cpm) of the CDS of all transcripts in Tm-treated cells were compared to untreated cells. List of significantly altered transcripts during Tm treatment in erythroblasts, cpm values are given for each sample for ribosome profiling and RNAseq.
(XLSX)

S3 Table List of upregulated transcripts during Tm treatment. Upregulated targets were uploaded on Genetrail2 to investigate enrichment of cellular component, biological processes and molecular function.
(XLSX)

S4 Table List of downregulated transcripts during Tm treatment. Downregulated targets were uploaded on Genetrail2 to investigate enrichment of cellular component, biological processes and molecular function.
(XLSX)

S5 Table Translation initiation sites detected by stalling of ribosomes in the presence of Harringtonine. Peaks were called with the cumulative reads of each triplicate, with our previously developed peak calling algorithm to identify translation initiation sites (TIS)[26]. Peaks were divided into 5’UTR TISs, annotated start codon TISs, TISs in the CDS, or in the 3’UTR. The analysis was performed both with a setting of peaks at -12nt and at -13nt from the read start. Peaks were assigned to AUG, CUG, GUG or UUG start codons at either +12 or +13 from the start of the protected fragment. All other peaks were assigned to the codon at the +13 position counted from the top of the peak. TISs in the 5'UTR, the CDS, annotated starts were fused to gene name in random order. Positions are +13 positions, unless a atg, ctg, gtg or ttg occurs at +12, or +14. in that case the atg, ctg, gtg or ttg was preferred.
(XLSX)

S6 Table Codons at -13 (P) position of translation initiation sites, measured after ribosome stalling with Harringtonine. Called peaks and triplet codons were compared in untreated and Tm-treated erythroblasts.
(XLSX)

S7 Table Transcripts with differential use of TIS in absence and presence of tunicamycin. Peak intensity ratio between TIS peaks in stressed cells were compared to untreated cells for specific transcripts. At a p-value less than 0.01 few transcripts revealed differentially employed TISs in their 5’UTR Coverage: cumulative reads of the peak. Statistics: two way ANOVA between triplicate samples of both conditions.
(XLSX)
